# Laser-Micro-Annealing of Microcrystalline Ni-Rich NCM Oxide: Towards Micro-Cathodes Integrated on Polyethylene Terephthalate Flexible Substrates

**DOI:** 10.3390/ma18030680

**Published:** 2025-02-04

**Authors:** Lara Ahrens, Martin Mikulics, Steffen Schröder, Joachim Mayer, Hilde Helen Hardtdegen

**Affiliations:** 1 Ernst Ruska-Centre, Forschungszentrum Jülich, 52425 Jülich, Germany; l.ahrens@fz-juelich.de (L.A.); j.mayer@fz-juelich.de (J.M.); 2 Central Facility for Electron Microscopy (GFE), RWTH Aachen University, 52074 Aachen, Germany; 3 Institute of Physical Chemistry and Center for Materials Research, Justus-Liebig-University Giessen, 35392 Giessen, Germany; steffen.schroeder@phys.chemie.uni-giessen.de

**Keywords:** Ni-rich NCM, micro-cathode, PET flexible substrate, Raman spectroscopy, laser-micro-annealing

## Abstract

Here in this work, we report on micro-Raman spectroscopy investigations performed on freestanding Ni-rich NCM (Li_x_Ni_0.83_Co_0.11_Mn_0.06_O_2_) microcrystals transferred to flexible polyethylene terephthalate (PET) host substrates. This technological procedure introduces a first building block for future on-chip-integrated micro-accumulators for applications in flexible optoelectronics, sensors, microbiology, and human medicine. An after-synthesis thermal treatment was used to help improve the material homogeneity and perfection of the cathode material. To this end, a local laser micro-annealing process was applied to the freestanding Ni-rich NCM microcrystals. The thermally initialized structural processes in the singular micro-cathode units were characterized and determined by micro-Raman spectroscopy. Micro-Raman mapping images revealed the evolution of a recrystallization process after the local annealing procedure. Furthermore, laser micro-annealing led to the suppression of the pristine “polycrystalline morphology” of the investigated micro-cathode regions. Besides the dominant characteristic Raman mode at ~1085 cm^−1^, most likely ascribed to lithium carbonate, metal oxides with Raman modes around ~550 cm^−1^ were identified. This highly efficient transfer and integration technology represents a basic building block towards micrometer-sized accumulators for a large range of emerging applications.

## 1. Introduction

Laser-assisted processing has become an important tool for the realization of various technological procedures applied in lithium-ion battery (LIB) research and developments, with the aims of improving/tuning material properties and increasing device efficiency, as well as reducing fabrication costs in mass production fabrication lines. These efforts were already demonstrated and reported over 2 decades in a large amount of publications [1,2,3,4,5,6,7,8,9,10,11,12,13,14,15,16,17] and systematically summarized in reviews by Pfleging [18,19]. Further progress in laser techniques applied to cutting, structuring, and printing of battery materials will be the precondition for the industrial realization of novel e.g., 3D battery architectures. In addition, further developments in complementary and correlative characterization techniques [6,20,21,22] will provide deep insights into material chemistry and physics and will achieve improved cycle stability and cell lifetime [3,18,23] for the development of next-generation LIB solutions.

In general, the healing of defects, as well as phase and structural engineering in LIB materials, seems to be a crucial point which requires sophisticated techniques, such as laser annealing down to the micrometer scale, as previously reported [2,3,6]. These developments are standardly accompanied by a number of non-destructive characterization tools, such as X-ray diffraction (XRD) [24], X-ray photon spectroscopy (XPS) [25], scanning electron microscopy (SEM) [26], electrochemical impedance spectroscopy [27], and nuclear magnetic resonance (NMR) [28], and some less-accessible methods, such as X-ray computed tomography [22] and neutron radiography [29]. They determine different important characteristics for the process of synthesis and structure formation, such as element distribution, chemical compound formed, and morphology. The techniques deliver information on different-length scales from the macroscale down to the micro- and nano-scales. For XRD and electron microscopy techniques, the determination of light elements, such as Li and H, is challenging/difficult. Spectroscopic techniques, on the other hand, such as Raman spectroscopy, among others, provide information on chemical compounds and binding. All in all, developments in characterization techniques, especially with respect to in situ and operando employments, can provide valuable insights into degradation mechanisms for LIB [30]. Due to its high spatial resolution and chemical specificity (also for lighter elements), micro-Raman spectroscopy has gained importance, especially for following chemical reactions. Lately, materials have been observed/characterized, if possible, in situ/and or in operando [31] during their employment at the (often high-temperature) conditions the materials are used. The special advantage is that the material can be characterized without any complex sample preparation [32,33,34,35].

These techniques and developments are necessary for the realization of future compact and reliable on-chip-integrated micrometer-sized accumulators for highly efficient micro- and nanometer-sized optoelectronic devices [36,37,38,39,40,41,42,43,44,45,46,47,48,49,50]. The small dimensions of the devices are a prerequisite to decrease the overall energy consumption for emerging technologies. Furthermore, biocompatibility, mechanical properties, and especially device flexibility during extremely large time periods, such as in the case of medical implants and other biological systems, are required. As a consequence, alternative micrometer-sized accumulator/LIB architectures have to be developed with respect to long-term device stability. Additionally, the operation under harsh environmental conditions has to be taken into account by the device design. Therefore, the predictable/expected material properties play an important role in device integration technologies. Furthermore, suitable material model systems, which also take into account possible device aging and corrosion, as well as degradation processes, are the basis for the future mass production of “real” devices. Here, in this work, we started with the development of a flexible device platform which could serve as the basis for highly efficient, biocompatible “energy”/rechargeable sources for driving a variety of micro- and nanometer-sized devices. They could serve not only in conventional applications but—as we mentioned above—also in emerging technologies, such as in neuro-optical interfaces, optical computing, and/or hardware core elements for artificial intelligence architectures. The development of these novel technologies is accompanied by efforts to achieve deep insights into novel materials and devices by exploring their physical, mechanical, chemical, and/or biological properties. We chose Ni-rich nickel cobalt manganese oxide (NCM) material as the basis for the investigations towards a future rechargeable power source.

Here, in this contribution, we introduced a first building block for future micrometer-sized accumulators based on flexible PET substrates. The key technology necessary for their realization is based on local laser micro-annealing (similar to techniques discussed above). As it has been demonstrated that local laser micro-annealing can enhance material properties [51], it was applied to the freestanding Ni-rich NCM microcrystals as well. The Ni-rich microcrystals were subjected to different laser power densities and annealing times. The pristine material was characterized by EDX, SEM, HAADF-STEM, and micro-Raman spectroscopy beforehand and directly after each annealing step by micro-Raman spectroscopy. By doing so, the studies helped bridge the length scale between global information obtained from X-ray diffraction (XRD) [52] studies and the local information on the nanoscale provided by transmission electron microscopy (TEM) investigations. An improvement in the material uniformity by the LMA process was demonstrated, which is extremely important for the micrometer size of the cathodes envisaged.

## 2. Materials and Methods

The materials investigated by Raman spectroscopy (Renishaw inVia FSM REFLEX, New Mills, Gloucestershire, UK) in this study were prepared by mixing NiO, Co_3_O_4_, MnO, and LiOH·H_2_O in a mortar with a Li: transition metal (TM) ratio of 1.1 to form NCM831106. The initial heating program comprised 6 h at 975 °C for the samples of microcrystals used in the Raman experiment and 875 °C for the sample used in the STEM-EDX analysis, followed by a further 6 h at 680 °C. The heating and cooling rates were 100 K/h, and the process was conducted under an oxygen flow of 100 sccm, which also applied to the consecutive heating steps. The NCM obtained from the initial heating phase was deagglomerated in a mortar and combined with Li_2_CO_3_ (Li:TM ratio of 0.8). The second heating program included 40 h at 750 °C, followed by 20 h at 680 °C. Subsequently, the material was ground and washed with water (cathode active material (CAM)): water ratio of 1 g:20 g) for 45 min in a beaker under stirring, with 1/5 of the water added as ice. A post-annealing step was then performed at 750 °C for 3 h. Finally, the material was deagglomerated once more in a mortar and sieved through a 40 µm mesh. A more detailed description of the synthesis procedure can be found in the article by Ruess et al. [52].

The Raman studies were carried out in a confocal Raman microscope (Renishaw) in backscattering geometry, which was equipped with a frequency-doubled Nd-YAG laser (532 nm) and a CCD detector. The backscattered signal was collected through a 100× objective lens and dispersed by a 2400 grooves per mm grating. The laser power was kept below 0.1 mW to avoid sample damage and any heating effects. The spectrometer was referenced to the transverse optical phonon of Si at 521 cm^−1^. Spectra were recorded in the range between 100 cm^−1^ and 2000 cm^−1^. The laser micro-annealing procedure was performed with the help of a HeCd continuous wave (cw) laser (325 nm, 25 mW). The Ni-rich NCM microcrystals (selected from the same preparation “batch”) were annealed at three different laser power densities and at different exposition times. After the microcrystals were exposed to the respective laser power density (325 nm)/annealing time, they were characterized by Raman spectroscopy (532 nm) at room temperature (RT) in a step-by-step mode. For the sake of statistics, six microcrystals were selected with almost identical pristine (i.e., non-annealed) material characteristics/spectra as a starting point for each annealing experiment series. The results were nearly identical for comparative series. This procedure led to very similar results. The LMA process proved to be a reproducible technique.

For scanning electron microscopy (SEM) imaging, the cathode powder was distributed on a carbon pad. The SEM images were acquired at 5 kV using an InLens detector (Zeiss, Oberkochen, Germany). For transmission electron microscopy (TEM) investigations, a lamella was prepared by a gallium-focused ion beam (FIB, Helios NanoLab 460F1, FEI). High-angle annular dark field (HAADF) scanning TEM images were obtained with an aberration-corrected TEM (Spectra 300, Thermo Fisher Scientific, Waltham, MA, USA) operated at 200 kV and equipped with an energy-dispersive X-ray detector Super X system (Thermo Fischer Scientific).

## 3. Results

**Figure 1:**
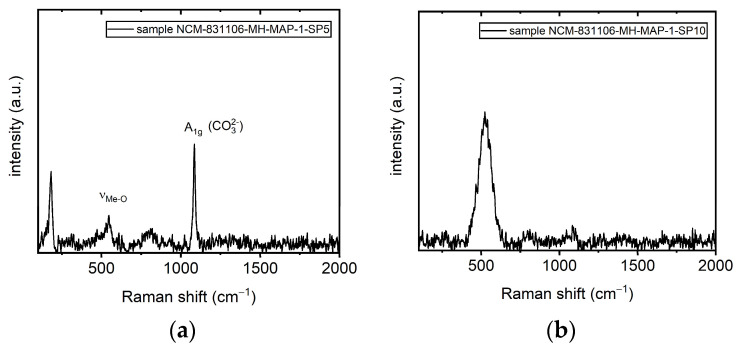
Two different representative Raman spectra (**a**,**b**) collected in the area under investigation of the NCM (pristine material) shown in Figure 2a,b at different magnifications.

The pristine as-prepared Ni-rich NCM material was transferred onto a chip-glass carrier and was characterized optically with the help of micro-Raman measurements as a reference before further studies were carried out with the material transferred to flexible polyethylene terephthalate (PET) host substrates. Representative Raman spectra are presented in Figure 1a,b. They revealed a strong inhomogeneous “fingerprint” of the investigated microcrystals across a large area. A comparison of both representative spectra revealed a large intensity difference/signature difference in the Raman mode at ~1085 cm^−1^. This mode was not found only once but reproducibly, as additional Raman measurements confirmed. This very prominent and narrow Raman mode was also observed for benzene [53] or calcite [54]. However, these compounds exhibited different Raman fingerprints. The mode, as well as the modes below 200 cm^−1^, can be attributed to lithium carbonate in the material under inspection [55,56,57,58]. On the other hand, a broad, singular Raman mode, ~520–580 cm^−1^, was also observed. In general, layered Ni-rich NCM crystallizing in the rhombohedral structure give rise to two Raman-active modes. They correspond to O-Me-O bending (E_g_) and Me-O stretching (A_1g_) vibrations [21,59,60]. The intensity and the form of these modes can be affected strongly by the experimental conditions, such as, among others, the laser wavelength [61], the laser power density, and also by the ambient they are studied in [62]. Furthermore, the shapes of the modes are affected by the NCM composition, since the bands consist of overlapping modes of the respective Ni-, Co-, and Mn-related vibrations with different Raman shift positions [59]. Reductions in the mode intensities are a strong indication for degradation/phase transition by cation mixing, at first towards the spinel phase and, upon further degradation, at last towards the cubic rock-salt phase, with an increasing intermixing of the Li and the metals (Ni, Co, and Mn) and the symmetry increase in the crystal structure [61]. This mode is electrochemically inactive and certainly not desired for LIB applications. An increase in the intensity of the modes, on the other hand, was reported to be related to a healing of defects at grain boundaries or an increase in grain sizes, for example, by recrystallization, reducing the disorder in the NCM towards a perfectly layered rhombohedral structure [61]. For future micrometer-sized cathode applications, the homogeneity of the material is especially important, which can be investigated by Raman spectroscopy as well. The uniformity of the LIB materials could then be demonstrated by a uniform intensity of the A_1g_ and E_g_ modes across the investigated microcrystal area. Any additional modes seen in the spectrum may be related to the formation of oxides of the transmission metals and also Li_2_CO_3_ [61], indicative of a recrystallization and a redistribution/migration of Li in the microcrystal. The loss of Li would be detrimental to electrochemical activity. However, it must be noted that Li_2_CO_3_ itself on the surface of Ni-rich NCM-positive electrodes is not detrimental to LIB performance if it is used as an (intentional) coating. Then, it can help improve cell cyclability and performance stability [63].

**Figure 2:**
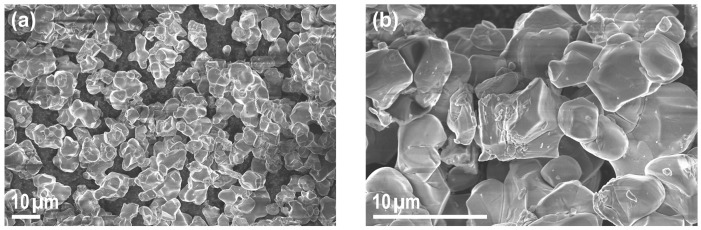
Detail SEM images at (**a**) 1000-fold magnification and (**b**) 4000-fold magnification of the investigated material - of “pristine” Ni-rich NCM microcrystals/powder.

The corresponding SEM images are presented in Figure 2a,b at different magnifications, showing a variety of morphologies, including non-single crystalline appearance and small grains or fragments on the surfaces of other particles.

**Figure 3:**
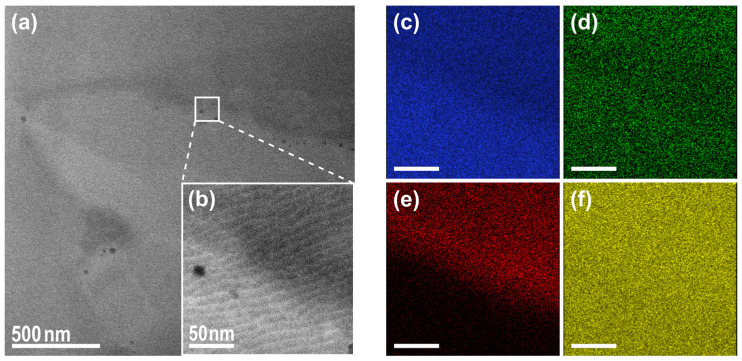
HAADF-STEM images of the Ni-rich NCM FIB lamella in an overview (**a**) and in a larger magnification of the highlighted white rectangular area (**b**). The corresponding EDX elemental maps are shown for the highlighted area for (**c**) nickel, (**d**) cobalt, (**e**) manganese, and (**f**) oxygen, revealing the inhomogeneity of the material, especially with respect to manganese distribution after calcination at 875 °C. In addition, a significant number of pores formed along the interface of manganese-rich and manganese-depleted regions.

TEM analysis of the same powder synthesis, with the difference being that the upper calcination temperature was at 875 °C instead of 975 °C, is shown in Figure 3. The higher calcination temperature was reported to have the effect of decreasing the off-stoichiometry, as reported by XRD studies, i.e., influencing the structural quality advantageously and, on the other hand, increasing the particle size [52]. The HAADF image clearly revealed the material’s inhomogeneity within a particle using the different contrasts, which were proportional to the atomic number Z. EDX measurements confirmed a manganese-depleted area, indicating that the MnO precursor did not fully react. It is beyond doubt that the calcination temperature represents a pivotal parameter in the synthesis process, and its influence should not be underestimated. Nevertheless, an examination of the Raman signal inhomogeneities provided substantial evidence that even at elevated calcination temperatures, significant defects in the chemical bonding remained undetected by alternative techniques, such as SEM or XRD.

**Figure 4:**
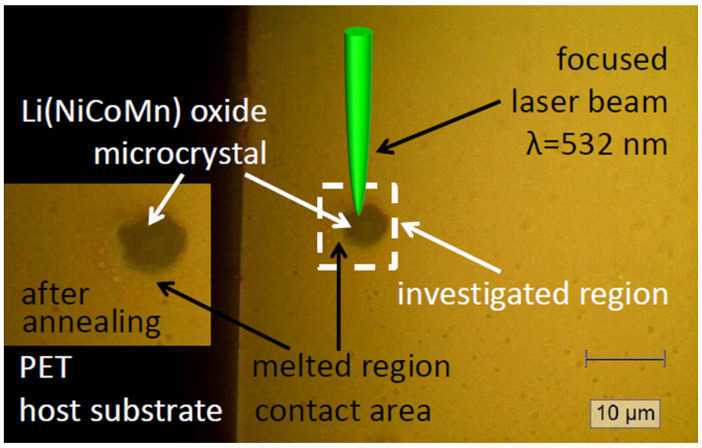
Optical images (after the laser micro-annealing process) and schematics of the micro-Raman measurements performed on the annealed NCM microcrystal. (Inset: detail of an annealed microcrystal and the melted region after the laser micro-annealing process). Representative Raman spectra are presented in the following section.

For the annealing experiments, all microcrystals were transferred to the host PET substrates with the help of the micropipette transfer technique already described in our previous work [64,65]. Mechanical stability was achieved by “melting” the PET locally around the microcrystal position and the subsequent annealing of the metal contacts. Here, it should be noted that in this study, we introduced only the first building block of an “energy”/rechargeable source: a unit with an integrated micro-cathode. Polyethylene terephthalate (PET) is cheap and an almost fully recyclable flexible material on which, in the last ~2 decades, a variety of devices were already realized and demonstrated, such as thin-film transistors [66], LEDs [67], displays [68], solar cells [69], sensors [70], picosecond photodetectors [71], and other applications [72]. Since it has a high surface resistivity (~10^13^ Ω/sq), withstands chemical corrosion, and is well known for its high optical transparency in the VIS range, it could be suitable in combination with a printed display technology for the mass production of numerous future applications used in daily life to substitute e.g., newspapers, easy-handling portable screens in offices, carrier mediums for drawings/schematics, etc. These products would also be well-suited for operation under harsh environments, e.g., in buildings or in a large range of production industries. Hence, it would be of benefit if such flexible substrates could be provided with micrometer-sized and compact batteries/accumulators. After transferring the microcrystals to the flexible host PET substrate, precise local annealing of the NCM microcrystal was performed by scanning the surface over the target region with the aim of initializing a recrystallization process. The laser micro-annealing experiments were carried out in a step-by-step procedure. After each annealing experiment (i.e., at the specified laser power density/annealing time), the respective microcrystal was studied by Raman spectroscopy, as presented schematically in Figure 4. It also shows an optical image of a laser micro-annealed micro-crystal.

**Figure 5:**
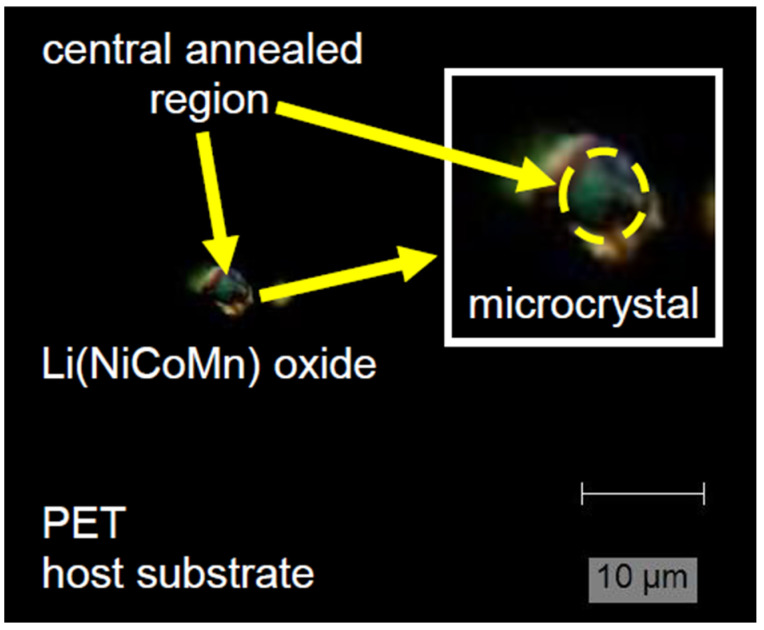
Representative microscope image of the transferred Ni-rich NCM microcrystal onto the PET substrate (after the laser micro-annealing process). The laser micro-annealing (LMA) process was tuned carefully with respect to the prevention of thermal damage.

**Figure 6:**
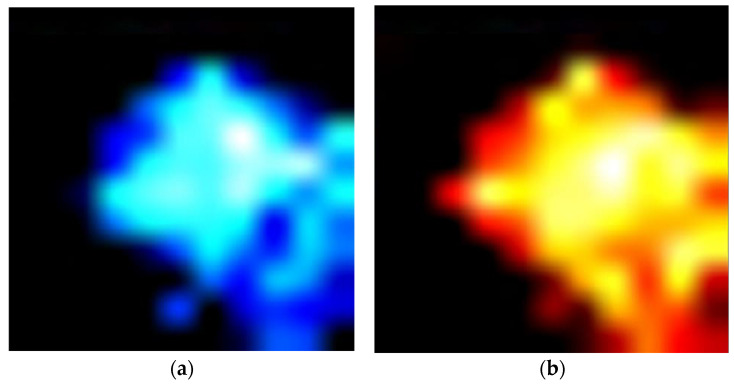
(**a**) Micro-Raman intensity mapping performed on the transferred Ni-rich NCM microcrystal at the Raman mode at 470 cm^−1^ and (**b**) at 550 cm^−1^, related to O-Me-O bending (E_g_) and Me-O stretching (A_1g_) vibrations [21,59,60], respectively.

The optical image (Figure 5) from a single microcrystal, transferred to the host PET substrate, revealed different “colors” across the whole visible crystal area, indicative of the formation of different metal oxides, e.g., MnO_2_ (red–brown, inset Figure 5), at the surface. Micro-Raman mappings carried out over the whole microcrystal area of the microcrystalline material after laser micro-annealing are presented in Figure 6. Both Raman modes at ~470 cm^−1^ and ~550 cm^−1^ can be attributed to O-Me-O bending (E_g_) and Me-O stretching (A_1g_) vibrations [21,59,60], modes related to characteristic vibrations of Ni-O in the lattice [59,73]. The broadness of these modes was related to the overlapping vibrations of Co-O and Mn-O in the lattice, which slightly increased the Raman shift towards higher wavenumbers (due to their low concentration), as these metals had a lower molecular weight. The intensity micro-Raman mapping images presented in Figure 6a,b indicate that the laser micro-annealing (LMA) process performed over the “crystal” area caused “structural” changes manifested by an almost uniform intensity in the central region. Nevertheless, the laser power density, as well as the annealing time, should be chosen carefully. In the suboptimal case, a high-power density can initialize irreversible structural changes and results in the thermal decomposition of the “annealed” microcrystalline material.

**Figure 7:**
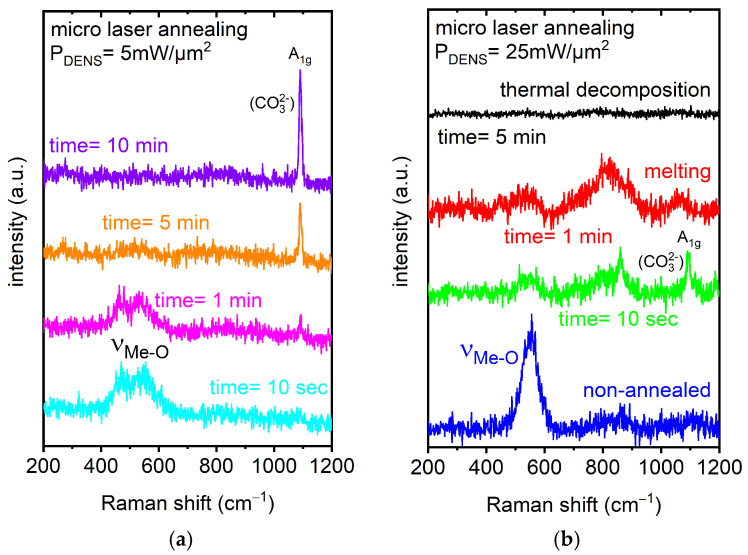
(**a**) Representative Raman spectra collected on the transferred Ni-rich NCM microcrystal exposed to a cw laser with an optical power density of ~5 mW/µm^2^ for 10 s and 1, 5, and 10 min on the microcrystal surface. (**b**) Raman spectra collected with an optical power density of ~25 mW/µm^2^ for 10 s and 1 and 5 min, compared with pristine, non-annealed material.

It could be expected that the application of a “lower” optical power density could be of benefit, since the LMA process can be performed without uncontrollable melting or thermal decomposition effects, which can occur abruptly. Hence, in the following, we demonstrated the evolution/development of Raman spectra for a “low” optical power density at ~5 mW/µm^2^ for 10 s, 1, 5, and 10 min, as shown in Figure 7a. The Raman spectra collected on the Ni-rich NCM microcrystal for a “low” optical power density (~5 mW/µm^2^) and at 10 s and 1 min were characterized by two modes. They can be attributed to O-Me-O bending (E_g_) and Me-O stretching (A_1g_) vibrations [21,59,60] of the layered rhombohedral structure. They developed—upon annealing—from the broad band observed for the pristine microcrystal. This indicated an improvement in the microcrystal towards the rhombohedral layered phase. The spectrum recorded after the annealing time increased from 10 s to 1 min, indicating that the intensity already decreased. This would be an indication for the beginning of a phase change to the spinel and, later, to the cubic rock-salt structure. A further increase in the annealing time led to the “suppression” of the dominant characteristic Raman modes, indicating an irreversible phase transformation. Furthermore, a Raman mode evolved at ~1085 cm^−1^, which, again, would be attributed to lithium carbonate. The intensity of this mode increased upon a further increase in the annealing time. This can be explained by the “recrystallization” in the regions. Nevertheless, further systematic analyses and modeling of the lithium migration and diffusion processes, as well as dynamics induced by the LMA technique/procedure, will be the key to the improvement of material and device design (novel architectures) towards “long-life” and stable performance micrometer-sized LIB/accumulator cell units.

The effect for a “border case”, when the thermal laser annealing treatment initialized the local melting of the Ni-rich NCM microcrystal, was clearly demonstrated by Raman spectra presented in Figure 7b. The “high” optical power density at ~25 mW/µm^2^ for 1 and 5 min was applied “step by step” to the microcrystal (presented in Figure 5) in the central region after the “low” power optical power density LMA process. It was evident that the intensity of the broad mode attributed to Me-O stretching (A_1g_) vibrations [21,59,60] began to decrease from 10 s to 1 min of annealing time. This decrease in intensity was already an indication of a phase transition. Furthermore, melting was also observed, which meant that the microcrystal`s structure lost its crystal symmetry. In addition, it was already reported that the laser annealing at high power densities in the ambient “air” induced further oxidation of the material [61]. Further Raman modes started to evolve. Therefore, there were indications that the microcrystal must also have changed chemically [62] as well. We also observed that the lithium carbonate-related Raman mode at ~1085 cm^−1^ increased for a 10 s annealing treatment and then broadened and decreased for a 1 min annealing time. This effect could be related to the “migration” of lithium from the annealed region by local “high” temperatures caused by the LMA process. Further complementary investigations are certainly necessary to unambiguously clarify the structural and chemical changes. These Raman spectra demonstrated a strong effect of annealing already on a “short” time scale for a “high” optical power density.

In contrast, an additional “short” 10 s annealing time at high optical power density (~25 mW/µm^2^), as presented in Figure 7b (green spectrum), resulted in an intensity decrease in the Raman mode at ~1085 cm^−1^ in comparison with the representative Raman spectrum (violet) presented in Figure 7a. For the sake of comparison, an overview of the identified characteristic Raman modes extracted from the presented data, together with the respective assignments in Table 1, is provided at the end of this section.

**Figure 8:**
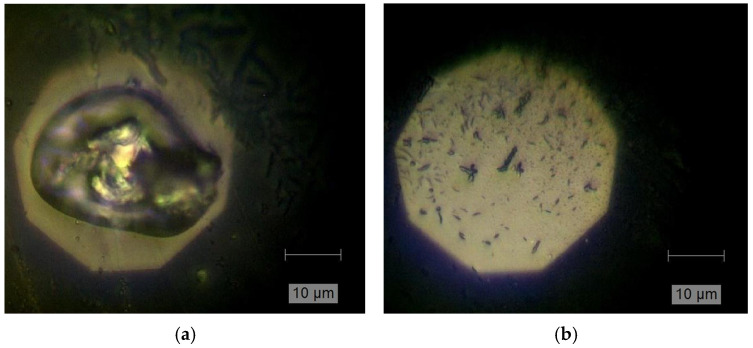
Representative optical images of the transferred Ni-rich NCM microcrystals exposed to a cw laser at an optical power density of ~25mW/µm^2^ for 1 min (**a**) and 5 min (**b**)**.** The respective Raman spectra collected in the central region are presented in Figure 7b and labeled as “melting” process and “thermal decomposition”.

The optical images presented in Figure 8a,b demonstrate the effect of “high” power optical densities applied to the transferred Ni-rich NCM microcrystals. Besides the melted crystal morphology visible in the central region with a diameter of around 10 micrometers, adjacent melted regions with polyethylene terephthalate compounds were identified and also confirmed by Raman spectra. This will be presented in our future studies. The formation of “melted” Ni-rich NCM oxide/polyethylene terephthalate was influenced by the micro-annealing process, as can be seen from the evolution studies of Raman modes on the Ni-rich NCM microcrystals presented in Figure 7b.

**Figure 9:**
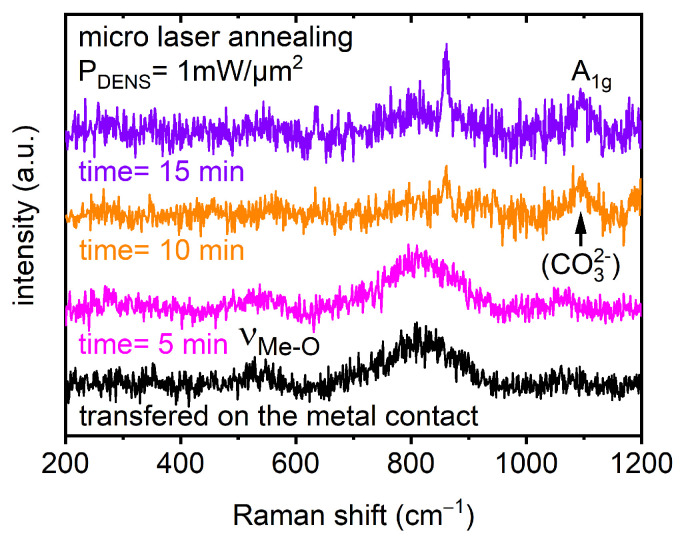
Representative Raman spectra collected in the central region of the Ni-rich NCM microcrystal transferred onto the metal contact (corresponding with the inset in Figure 4 presenting the detail of the annealed microcrystal). The laser micro-annealing process was performed at total annealing times of 5, 10, and 15 min and at a constant laser power density of 1 mW/µm^2^.

Finally, Figure 9 presents Raman spectra collected on the Ni-rich NCM microcrystal transferred directly onto a metal (Au) electrode evaporated on the polyethylene terephthalate flexible substrate. This step represents a first building block for future on-chip-integrated micrometer-sized accumulators. Details of the optical image of the transferred Ni-rich NCM microcrystal are presented in Figure 4, demonstrating the “micro-Raman characterization” procedure schematically. Micro-Raman measurements, performed on the microcrystal exposed at “low” optical power densities (~1 mW/µm^2^), revealed a strong impact of the LMA procedure on the timescale between 5 and 15 min. The intensity of the characteristic Raman mode at ~1085 cm^−1^, related to lithium carbonate, increased with the annealing time (Figure 9). This can be a result of the “migration” or the “diffusion” process of lithium ions or of the recrystallization of the microcrystal structure. Nevertheless, further systematic analyses and modeling of the lithium migration and diffusion processes, as well as dynamics induced by the LMA technique/procedure, will be the key to the improvement of material and device design (novel architectures) towards “long-life” and stable performance micrometer-sized LIB/accumulator cell units. 

Furthermore, a broad Raman mode was detected in the range between 770–890 cm^−1^. Additional investigations are necessary to clarify if the underlying Au contact area affected the material properties and, therefore, the material/structural properties recorded for Ni-rich NCM microcrystals.

An overview of the ex situ Raman spectroscopic results of NCM microcrystals transferred onto the PET flexible substrate is presented in Table 1. It becomes clear that investigations with further complementary and correlative characterization techniques dedicated to this task are necessary in the future to unambiguously attribute our findings to the degradation pathways already published. Then, a deeper understanding of the processes behind the observed effects of laser micro-annealing on the vibrational properties can be achieved, and the origin/nature of the physical and/or chemical mechanisms can be revealed. These studies are, however, beyond the scope of this report.

## 4. Conclusions

In this study, we introduced a first building block towards future micrometer-sized LIB/accumulators on flexible substrates. We transferred Ni-rich NCM microcrystals directly onto polyethylene terephthalate flexible substrates and, for the sake of comparison, onto a metal (Au) electrode. Micro-Raman investigations revealed the “positive” effect of the laser micro-annealing procedure on the development of a characteristic Raman mode at ~1085 cm^−1^, related to lithium carbonate if the “thermal” damage threshold for the investigated material can be avoided. In the case of microcrystals transferred directly onto the polyethylene terephthalate flexible substrate, the “critical” optical power densities were reached at ~25 mW/µm^2^ for 1 and 5 min. Both these stages were characterized by irreversible structural and chemical composition changes and manifested as “melting” and “thermal decomposition” processes. On the other hand, “low” power densities in the range from ~1 to 5 mW/µm^2^ exposing microcrystals during a “time window” of “several” minutes led to a significant increase in Raman intensity at ~1085 cm^−1^, which was an indicator of the presence of lithium carbonate in the investigated microcrystals. The results confirmed that the careful choice of LMA conditions (annealing time and incident optical power density) can be of benefit for the development of next-generation micrometer-sized accumulators/batteries for flexible opto-/electronic applications.

## Figures and Tables

**Table 1:** Summary of ex situ Raman spectroscopic results of NCM microcrystals transferred onto the PET flexible substrate from Figure 7a,b.

Optical Power Density (mW/µm^2^)	Annealing Time	Raman RT Study 200–1200 (cm^−1^)		Notice	Interpretation
		Mode	Assignment		
pristine	pristine	550	ν_Me-O_	broad band FWHM ~70cm^−1^	overlapping Me-O stretching and bending vibrations related to Ni, Co, and Mn as the metal (Me) [59,60]
5	10 s	470	E_g_		overlapping O-Me-O bending vibrations related to Ni, Co, and Mn as the metal (Me) [59,60]
		550	A_1g_		overlapping Me-O symmetric stretching vibrations related to Ni, Co, and Mn as the metal (Me) [59,60]
	1 min	470	E_g_	the intensity began to decrease	
		550	A_1g_	the intensity began to decrease	
		1085	A_1g_	appeared	CO_3_^2−^ symmetric stretching vibrations [55,57,58]
	5 min	470, 550	E_g_ and A_1g_	both bands: the intensity dramatically decreased	beginning phase transition
		1085	A_1g_	intensity increase	
	10 min	1085	A_1g_	intensity increase	recrystallization
25	10 s	520–580		several overlapping modes detected	
		860	2TO	intensity increase	Ni oxide-related [74]
		1085	A_1g_	intensity decrease	CO_3_^2−^ symmetric stretching vibrations [55,57,58]
	1 min	520–580		several overlapping modes detected	
		770–890		several overlapping modes detected	Ni oxide-related [74] melting process confirmed by optical inspection
		1050–1090	A_1g_	the intensity began to dramatically decrease	
	5 min	bands not detected anymore			thermal decomposition

## Data Availability

The original contributions presented in this study are included in the article. Further inquiries can be directed to the corresponding authors.
